# Curiosa in fine needle aspiration biopsy of the thyroid gland

**DOI:** 10.1186/1756-6614-6-S2-A56

**Published:** 2013-04-05

**Authors:** Stanisław Sporny

**Affiliations:** 1 Department of Dental Pathology, Medical University of Lodz, Poland

## 

The great importance of cytological examination in diagnostic procedures of thyroid gland pathological lesions is widely known. Unfortunately, cytology as a diagnostic method presents many limitations due to possible presence of elements abnormal for thyroid gland physiology and histology. Especially difficult in cytological diagnosis appear: among non-neoplastic lesions - dyshormonogenetic and amyloid goiter and Riedel disease and among neoplastic lesions - rare microscopic variants of tumours, neoplasms arising from ectopic tissues and nonepithelial tumours.

